# Recurrent Gastrointestinal Bleeding in an HIV-Positive Patient: A Case Report

**DOI:** 10.7759/cureus.10688

**Published:** 2020-09-27

**Authors:** Arda Yavuz, Ayşe Nur Toksöz Yıldırım, Kübra Akan, Yaşar Çolak, İlyas Tuncer

**Affiliations:** 1 Department of Gastroenterology, Istanbul Medeniyet University, School of Medicine, Istanbul, TUR; 2 Department of Pathology, Istanbul Medeniyet University, School of Medicine, Istanbul, TUR

**Keywords:** kaposi sarcoma, gastrointestinal bleeding, hiv

## Abstract

HIV is a global epidemic that needs a multidisciplinary approach. Gastrointestinal bleeding is uncommon in HIV-positive patients. In cases such as bacillary angiomatosis, Kaposi sarcoma, herpes simplex, histoplasmosis, and cytomegalovirus (CMV) colitis, the underlying reason could be HIV. The reason could also be unrelated to HIV, such as peptic ulceration, esophageal varices, and Mallory-Weiss. In our case, we report a patient who was admitted to the hospital three times. In the first admittance, he indicated using multiple nonsteroidal anti-inflammatory drugs (NSAIDs); however, we could not find the bleeding focus. He underwent surgery, at which time we detected a Kaposi sarcoma.

## Introduction

Kaposi sarcoma (KS) is a rare angioproliferative malignancy that can occur as a result of human herpes virus-8 (HHV-8) coinfection in HIV-positive patients. Patients with KS generally present with mucocutaneous lesions, although visceral and other extracutaneous involvements are possible. Gastrointestinal KS is generally asymptomatic (75%). Symptomatic patients may have abdominal discomfort, abdominal cramps, nausea, diarrhea, or bleeding. Diagnosis can be confusing because of the possibility of alternative etiologies. Even in the antiretroviral drug era, KS should always be considered as a potential diagnosis.

## Case presentation

**Figure 1:**
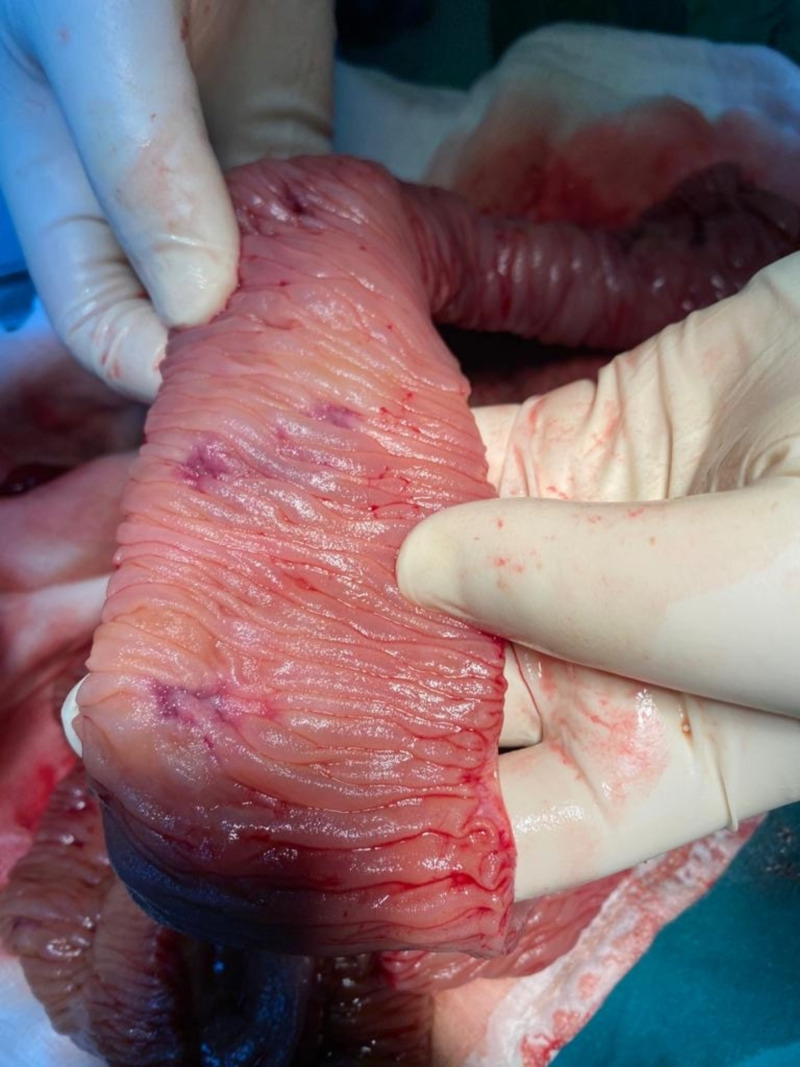
Macroscopic image 1 Vascular lesions in the small intestine

**Figure 2:**
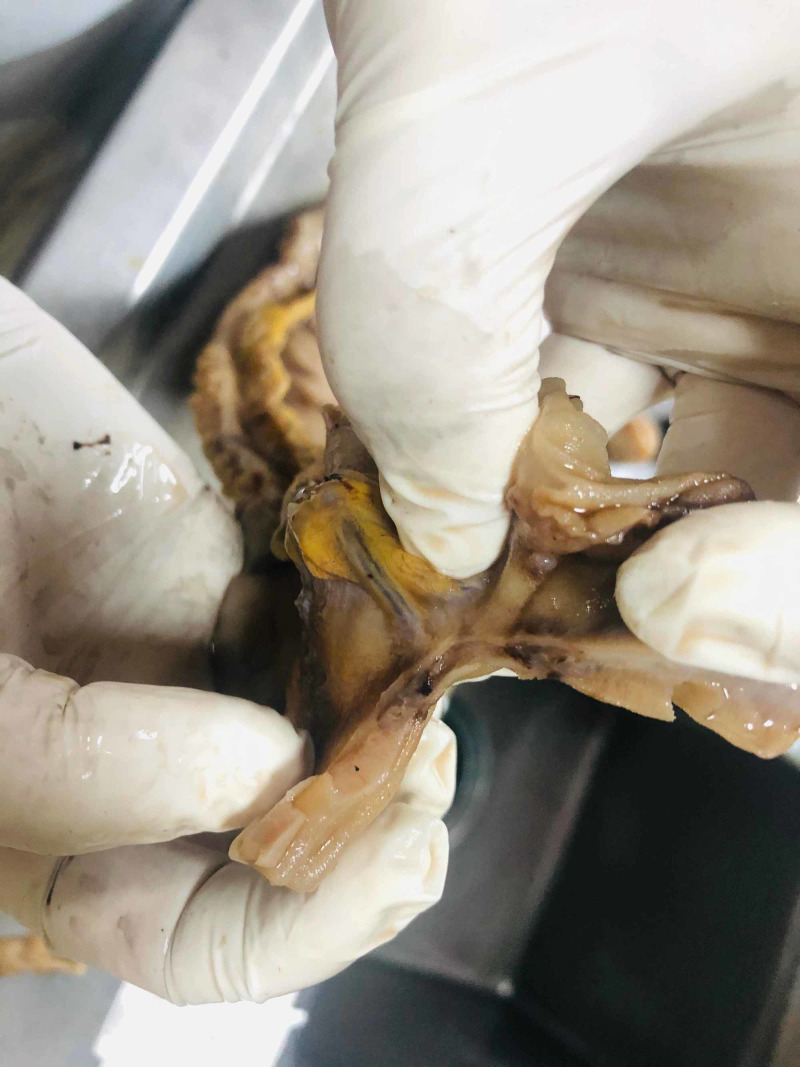
Macroscopic image 2 Reddish-brown to purple polyps and macules were seen, typically 0.5 – 1.5 cm

**Figure 3:**
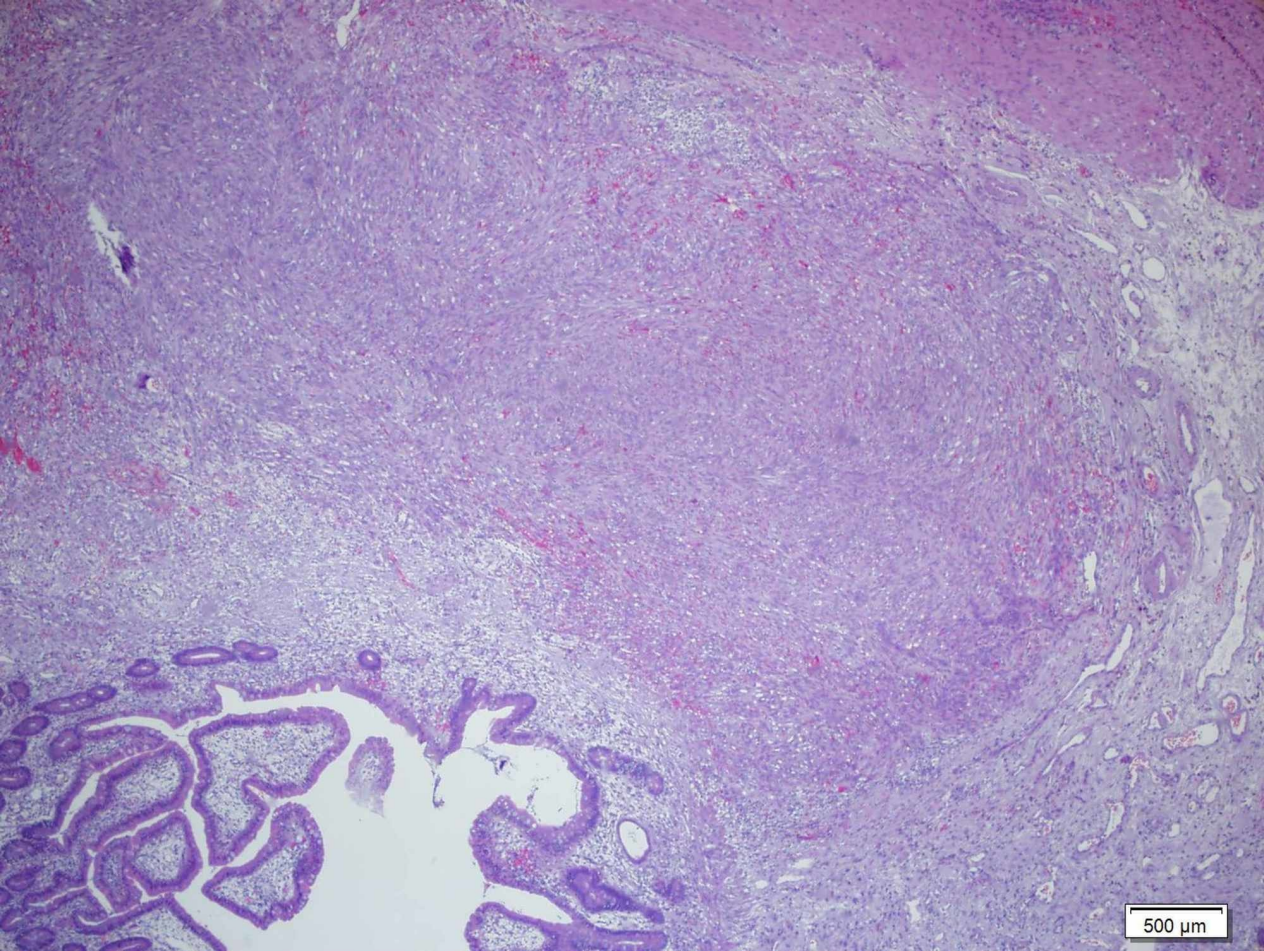
Hematoxylin and eosin stain Hematoxylin and eosin stain showing small intestine submucosa attacked by Kaposi sarcoma (H&E x100).

**Figure 4:**
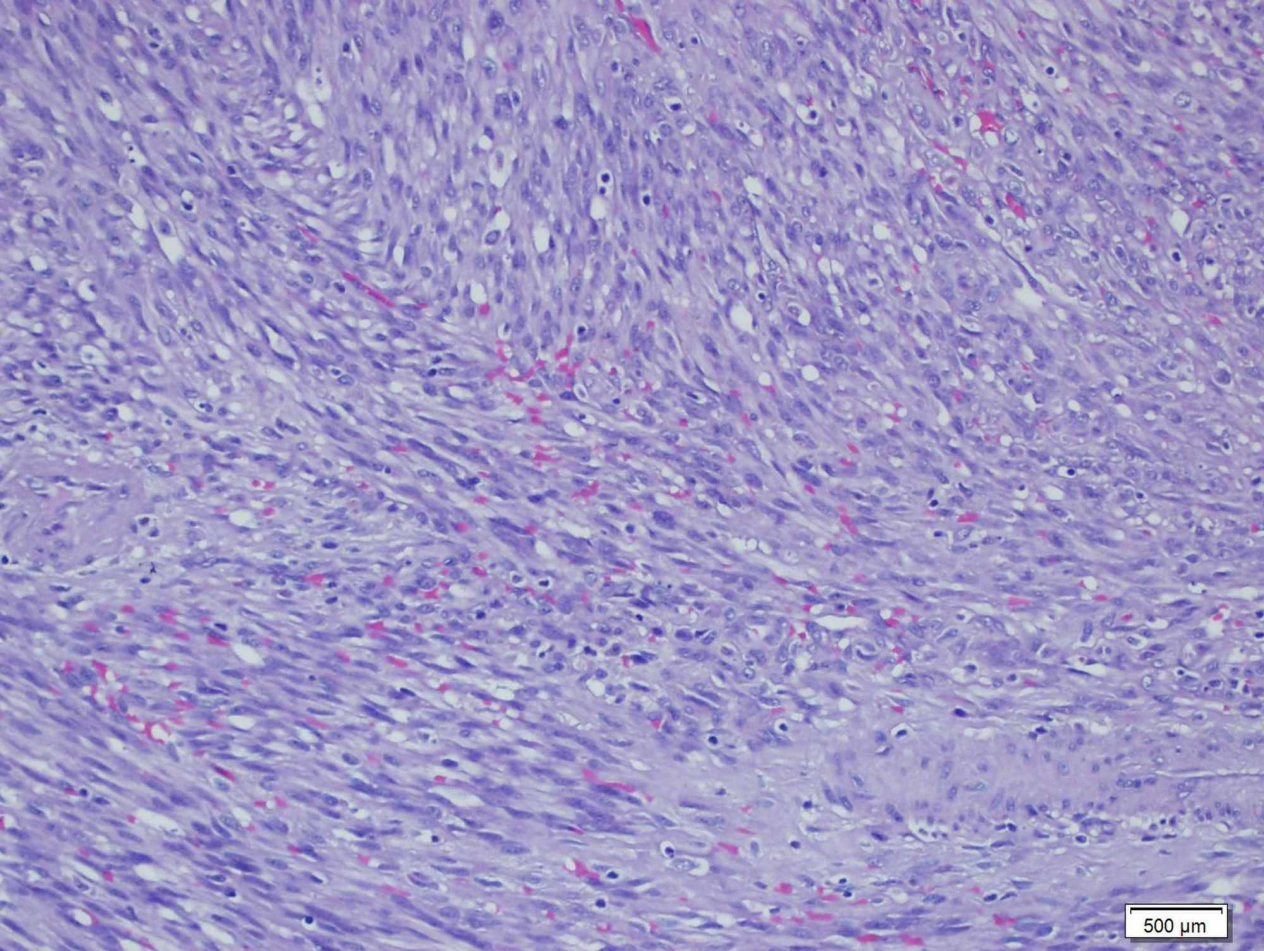
Hematoxylin and eosin stain Slit-shaped vessels filled with erythrocytes and lined with spindle-shaped cells (H&E x200).

**Figure 5:**
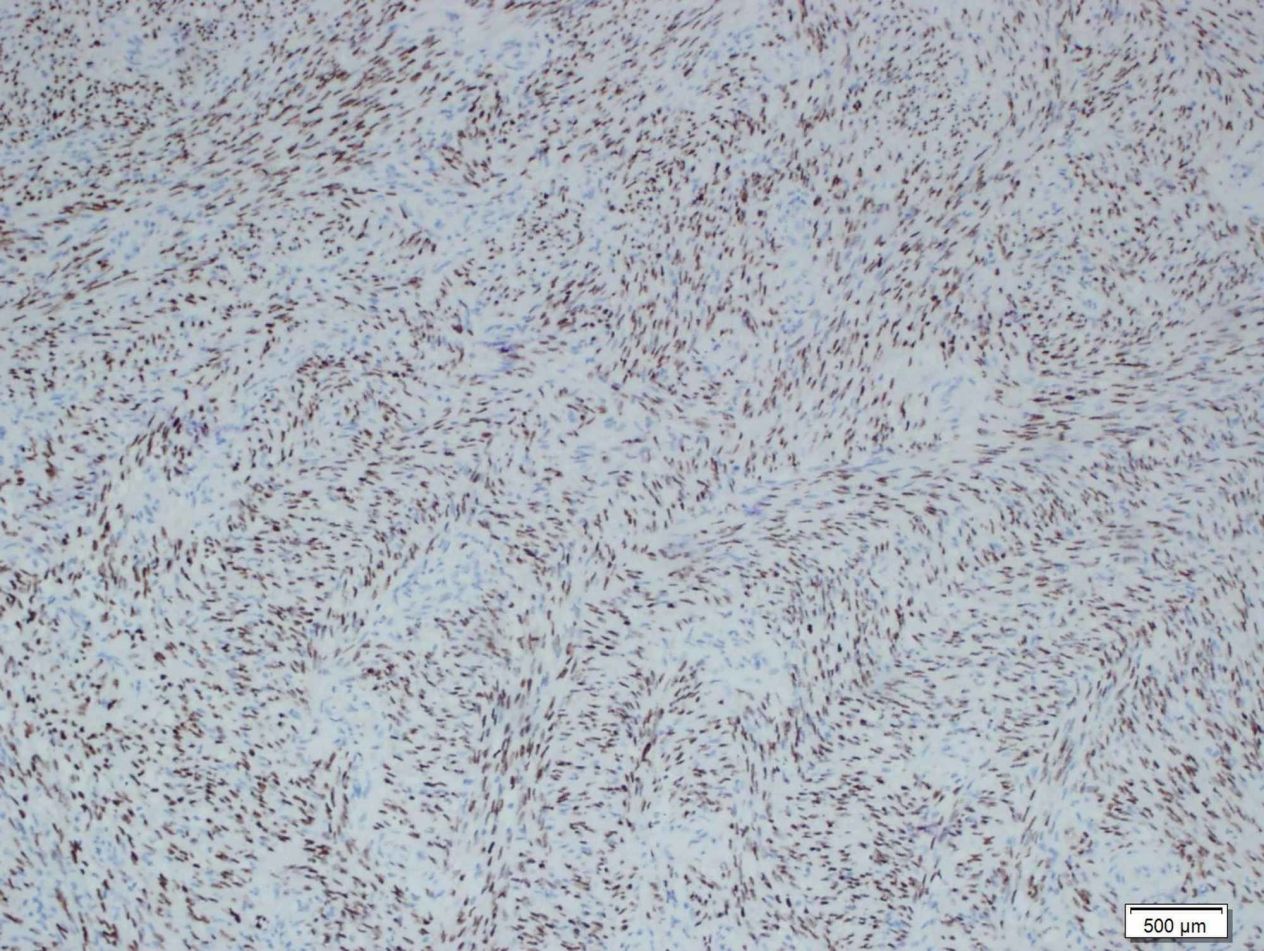
Immunohistochemistry The tumor cells react positively for human herpes virus-8 (HHV-8) antibody (x100)

**Figure 6:**
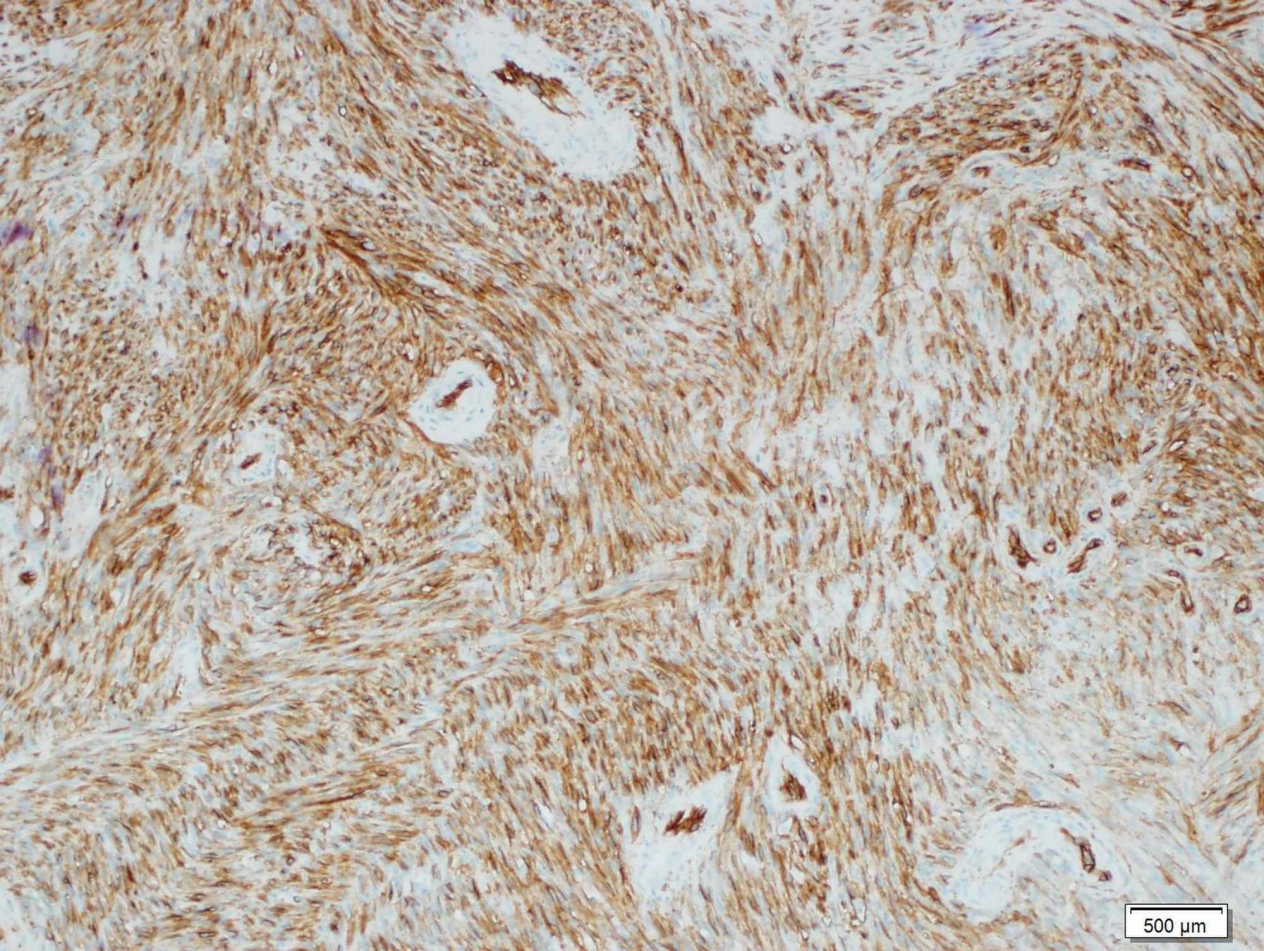
Immunohistochemistry The tumor cells react positively for CD34 antibody (x100)

**Figure 7:**
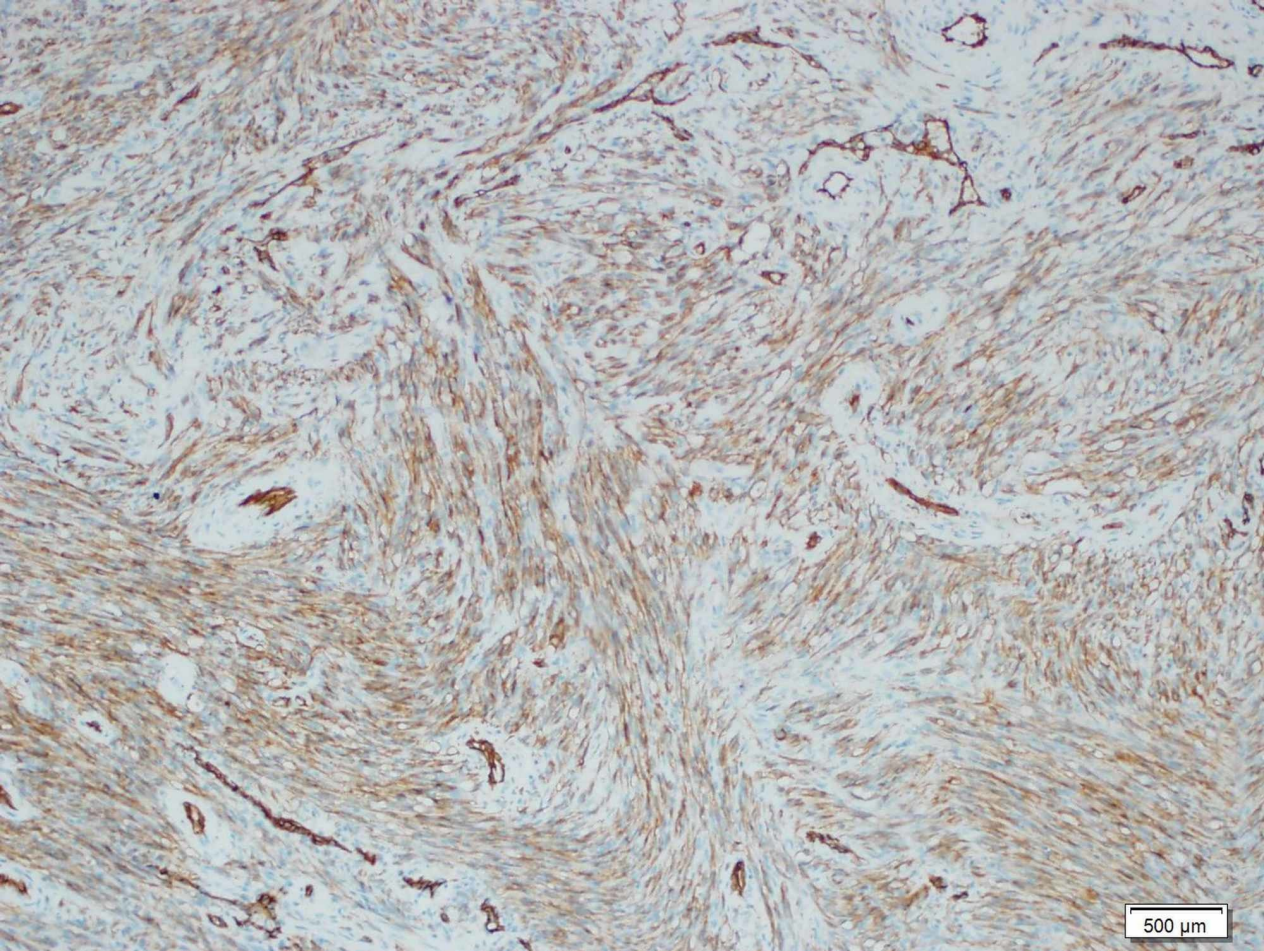
Immunohistochemistry The tumor cells react positively for CD31 antibody (x100)

A 55-year-old Turkish male was admitted to the hospital with melena. He had been HIV(+) for 10 years, but he was unfollowed. He complained of lumbar pain and, because of this, he used 15 tablets of nonsteroidal anti-inflammatory drugs (NSAIDs) per day. His creatine and potassium levels were mildly elevated. After hydration, his laboratory parameters became normal. His CD4 level was 7. The patient started tenofovir, emtricitabine, and dolutegravir, regarding infection with AIDS. On gastroscopy, there were clean-based erosive lesions but no active bleeding foci. The patient was discharged with the recommendation of the discontinuing of NSAIDs. After three weeks, he was readmitted and once again demonstrated no focus on repeat gastroscopy. The patient said that, because of the lumbar disk hernia, he had to use an NSAID. We prescribed tramadol and discharged him. Two weeks later, he experienced bleeding again, and his hemoglobin (Hb) level was 6 g/dL and blood pressure 90/60 mmHg. We performed gastroscopy and detected no focus; however, at this time he had hematochezia, and his Hb level did not increase despite the transfusions. We performed a colonoscopy which revealed blood in the lumen and colonic surface. We gave him infusions up to 20 U erythrocyte suspension over four days but could not increase his Hb level or stop the bleeding. We performed CT angiography but could not find any bleeding focus. Since capsule endoscopy and double-balloon enteroscopy were not available, we decided to perform laparotomy to exclude possible small intestinal bleeding sites. In laparotomy, multiple vascular lesions were observed (Figures 1, 2). A resection was performed. In the pathology specimen, the submucosal lesion was composed of spindle cells with mild to moderate atypia, arranged in vague fascicles, and separated by slit-like vessels (Figures 3, 4). Extravasated red blood cells were common. Immunostain for HHV-8, CD34, and CD31 were positive, supporting the above diagnosis of Kaposi sarcoma (Figures 5, 6, 7). He had no skin lesions. He was referred to oncology for further treatment planning. 

## Discussion

KS is a vascular neoplasm that includes four variants with different epidemiologic characteristics: a classic or sporadic form, an endemic form (African), an epidemic form associated with HIV and acquired immune deficiency syndrome, and an iatrogenic form associated with immunosuppressants. At the beginning of the HIV era, KS was seen in up to 50% of HIV-positive patients. In the era of highly active antiretroviral therapy (ART), the incidence of KS has decreased to less than 1% in HIV-infected patients. Cutaneous involvement is the most frequent manifestation across all four forms. Gastrointestinal KS is more common in the small bowel and stomach than in the colon or esophagus. Presentation varies from constitutional symptoms to epigastric pain, weight loss, and appetite. Most of the lesions are asymptomatic. Gastrointestinal bleeding, protein-losing enteropathy, diarrhea, intussusception, intestinal obstruction, or perforation can also be seen [1]. Endoscopic visualization can be helpful for diagnosis. A submucosal mass or red-purple color mass is typical for KS. The most common radiological finding in the gastrointestinal tract is multiple submucosal masses with or without a central ulceration (‘target’ or ‘bull’s eye‘ lesions). Plaque-like lesions or small nodules are rare manifestations [2]. On histopathology, KS is characterized by spindle cell proliferation, forming irregular vascular channels in the submucosal layer. An immunohistochemical stain for HHV-8 is recommended for diagnostic confirmation [3,4]. The first therapy for moderate skin lesions is highly active antiretroviral therapy. It can be sufficient to reduce the size of lesions; in 35% of cases, complete regression can occur within three to nine months of treatment. For rapidly progressive diseases such as extensive skin lesions or visceral involvement, pegylated liposomal doxorubicin (PLD), daunorubicin citrate liposome (DNX), adriamycin-bleomycin-vincristine (ABV), bleomycin-vincristine (BV), paclitaxel, and interferon-alpha are treatment options. For patients with limited skin lesions, local treatment with alitretinoin, vincristine, vinblastine, or sodium tetradecyl sulfate (STS) can be beneficial [5].

## Conclusions

This case involved an unfollowed patient with a HIV infection. He had a history of using ulcerogenic drugs, which made the case challenging; however, as a result, he was diagnosed with KS with small intestine involvement. There were no lesions in the upper and lower endoscopies. KS should be considered as a potential etiology of gastrointestinal bleeding, especially in HIV-positive patients.
